# Bayesian networks established functional differences between breast cancer subtypes

**DOI:** 10.1371/journal.pone.0234752

**Published:** 2020-06-11

**Authors:** Lucía Trilla-Fuertes, Angelo Gámez-Pozo, Jorge M. Arevalillo, Rocío López-Vacas, Elena López-Camacho, Guillermo Prado-Vázquez, Andrea Zapater-Moros, Mariana Díaz-Almirón, María Ferrer-Gómez, Hilario Navarro, Paolo Nanni, Pilar Zamora, Enrique Espinosa, Paloma Maín, Juan Ángel Fresno Vara

**Affiliations:** 1 Biomedica Molecular Medicine SL, Madrid, Spain; 2 Molecular Oncology & Pathology Lab, Institute of Medical and Molecular Genetics-INGEMM, La Paz University Hospital-IdiPAZ, Madrid, Spain; 3 Operational Research and Numerical Analysis, National Distance Education University (UNED), Madrid, Spain; 4 Biostatistics Unit, La Paz University Hospital-IdiPAZ, Madrid, Spain; 5 Functional Genomics Centre Zurich, University of Zurich/ETH Zurich, Zurich, Switzerland; 6 Medical Oncology Service, La Paz University Hospital-IdiPAZ, Madrid, Spain; 7 Biomedical Research Networking Center on Oncology-CIBERONC, ISCIII, Madrid, Spain; 8 Department of Statistics and Operations Research, Faculty of Mathematics, Complutense University of Madrid, Madrid, Spain; Fondazione IRCCS Istituto Nazionale dei Tumori, ITALY

## Abstract

Breast cancer is a heterogeneous disease. In clinical practice, tumors are classified as hormonal receptor positive, Her2 positive and triple negative tumors. In previous works, our group defined a new hormonal receptor positive subgroup, the TN-like subtype, which had a prognosis and a molecular profile more similar to triple negative tumors. In this study, proteomics and Bayesian networks were used to characterize protein relationships in 96 breast tumor samples. Components obtained by these methods had a clear functional structure. The analysis of these components suggested differences in processes such as mitochondrial function or extracellular matrix between breast cancer subtypes, including our new defined subtype TN-like. In addition, one of the components, mainly related with extracellular matrix processes, had prognostic value in this cohort. Functional approaches allow to build hypotheses about regulatory mechanisms and to establish new relationships among proteins in the breast cancer context.

## Introduction

Breast cancer is one of the most prevalent cancers in the world [1]. In clinical practice, breast cancer is classified according to the expression of hormonal receptors (estrogen or progesterone) and Her2, into positive hormonal receptor (ER+), HER2+ and triple negative (TNBC). In previous studies, our group defined a new ER+ molecular subgroup, named TN-like, with a molecular profile and a prognosis more similar to TNBC tumors [2]. We denoted the remaining ER+ tumors as ER-true. We also found significant molecular differences among breast cancer subtypes. For instance, differences related with metabolism of glucose were described between ER-true, TN-like and TNBC tumors [2, 3].

Proteomics supplies complementary information to genomics experiments. Proteomics has been used to find differences between subtypes on a proteomic level in sporadic and hereditary breast tumors [4]. In addition, proteomics coupled with super-SILAC has been also used to define molecular signatures that are differentially expressed between breast cancer subtypes [5].

Proteomics provides useful information about biological process effectors and may quantify thousands of proteins. Undirected probabilistic graphical models (PGM), based on a Bayesian approach, allow characterizing differences between tumor samples at functional level [2, 3, 6, 7]. In this study we explored the utility of Bayesian networks in the molecular characterization of breast cancer. The main feature of targeted Bayesian networks is that they provide a hierarchical structure and targeted relationships between proteins.

Bayesian networks (BN) have been previously used to inference protein signaling networks using phase-reverse protein array data from a breast cancer cell line [8]. In this study, the authors also experimentally tested some of the relationships by an inhibition approach. Baladandayuthapani et al. also applied BN to phase-reverse protein array data, in this case from a panel of ovarian and breast cancer cell lines. Their model was capable to distinguish between both cell line types [9] Most recently, BN has been used to determine genes related to bone metastasis development in breast cancer [10]. Also related to gene expression, BN inference leaded to the identification of TRIB1 as a regulator of cell cycle progression and survival in triple negative cancer cells [11]. BN have been also used to suggest therapeutic targets in breast cancer. In the study of Vundavilli et al., applying BN to gene expression data, the resulting network was used to rank different interventions in order to achieve an apoptosis induction [12]. Beretta et al. used BN to study the inference of signaling downstream of tyrosine kinase receptors, comparing predictions about inhibition of several nodes with experimental data [13]. BN was applied even to rank treatments in triple negative breast cancer datasets [14]. Finally, BN have demonstrated its utility in making associations between clinical data in breast cancer patients [15] or between lifestyle factors in breast cancer survivors [16].

In this work, we aim to explore if BN can be applied to proteomics expression data and if that the results provided by these analyses provide useful biological and clinical information. For this, we used mass-spectrometry proteomics data and Bayesian networks to characterize protein relationships in a cohort of breast cancer tumor paraffin samples. These networks maintained a functional structure and some of them showed prognostic value. This approach also reflected previously described protein-protein interactions and it could be used to propose new hypotheses and mechanisms of regulation of these proteins.

## Materials and methods

### Ethics statement

Written informed consent had been obtained for the participants on the study. The approval of the study was obtained from Hospital Doce de Octubre and Hospital Universitario La Paz Ethics Committees.

### Samples

One hundred and six FFPE samples from patients with breast cancer were recovered from I+12 Biobank and from IdiPAZ Biobank, both integrated in the Spanish Hospital Biobank Network. The histopathological characteristics were reviewed by a pathologist to confirm tumor content. Samples had to include no less than half of tumor cells. The endorsement of the study was obtained by Hospital Doce de Octubre and Hospital Universitario La Paz Ethics Committees. These samples were utilized in previous studies [2, 3, 17].

### Protein preparation

Proteins were extracted from formalin-fixed paraffin-embedded (FFPE) samples as previously described [18]. Briefly, FFPE sections were deparaffinized in xylene and washed twice with absolute ethanol. Protein extracts from FFPE samples were set up in 2% SDS buffer using a protocol based on heat-induced antigen retrieval. Protein concentration was quantified using the MicroBCA Protein Assay Kit (Pierce-Thermo Scientific). Protein extracts (10 μg) were processed with trypsin (1:50) and SDS was removed from digested lysates using Detergent Removal Spin Columns (Pierce). Peptide samples were additionally desalted using ZipTips (Millipore), dried, and resolubilized in 15 μL of a 0.1% formic acid and 3% acetonitrile solution before mass-spectrometry (MS) experiments.

### Label-free proteomics

Samples were analyzed on a LTQ-Orbitrap Velos hybrid mass spectrometer (Thermo Fischer Scientific, Bremen, Germany) coupled to NanoLC-Ultra system (Eksigent Technologies, Dublin, CA, USA) as described previously [2, 3]. Briefly, after separation, peptides were eluted with a gradient of 5 to 30% acetonitrile in 95 minutes. The mass spectrometer was operated in data-dependent mode (DDA), followed by CID (collision-induced dissociation) fragmentation on the twenty most intense signals per cycle. The acquired raw MS data were processed by MaxQuant (version 1.2.7.4) [19], followed by protein identification using the integrated Andromeda search engine [20]. Briefly, spectra were searched against a forward UniProtKB/Swiss-Prot database for human, concatenated to a reversed decoyed fasta database (NCBI taxonomy ID 9606, release date 2011-12-13). The maximum false discovery rate (FDR) was set to 0.01 for peptides and 0.05 for proteins. Label free quantification was calculated on the basis of the normalized intensities (LFQ intensity). Quantifiable proteins were defined as those detected in at least 75% of samples in at least one type of sample (either ER+ or TNBC samples) showing two or more unique peptides. Only quantifiable proteins were considered for subsequent analyses. Protein expression data were log2 transformed and missing values were replaced using data imputation for label-free data, as explained in [21], using default values. Finally, protein expression values were z-score transformed. All the mass spectrometry raw data files acquired in this study may be downloaded from Chorus (http://chorusproject.org) under the project name Breast Cancer Proteomics.

### Network construction

PGM are graph-based representations of joint probability distributions where nodes represent random variables and edges (directed or undirected) represent stochastic dependencies among the variables. In particular, we have used a type of PGM called Bayesian networks (BN) [13]. With these models, the dependences between the variables in our data are specified by a directed acyclic graph (DAG). The obtained networks will indicate causality i.e. if protein A and B are connected and protein A changes its expression value, protein B changes its expression value as well [22].

Firstly, we find the BN that best explains our data [23]. There are different algorithms to learn a DAG from data but we have selected the well-known PC algorithm (named as its inventors Peter Spirtes and Clark Glymour), a constraint-based structure learning algorithm [24] based on conditional independence tests. The PC algorithm was shown to be consistent in high-dimensional settings [25]. Moreover, an order-independent version of the PC algorithm, called PC-stable, was proposed in [26]. All these procedures are implemented in R within packages pcalg [25] and graph [27]. We used protein expression data without other a priori information.

In this way, our data are represented by a large graph that can be partitioned into several connected components. Then, we focused on finding suitable subgraphs that give us a much clearer understanding of the interrelations therein.

STRING v11 (https://string-db.org/) was used to check if some of the protein relations obtained in the DAG analysis were previously described.

### Gene ontology analyses

Protein to Gene Symbol conversion was performed using Uniprot (www.uniprot.org) and DAVID (www.david.ncifcrf.gov) [28]. Gene Ontology Analysis was also done in DAVID selecting only “Homo sapiens” background and GOTERM-FAT (http://geneontology.org/), Biocarta (http://doi.org/10.1089/152791601750294344) and KEGG (https://www.genome.jp/kegg/kegg1.html) databases.

### Component activity measurements

Component activities were calculated as previously described [2, 3]. Briefly, activity measurement was calculated by the mean expression of all the proteins of each component related with the established major component function.

### Statistical analyses

Network visualization was performed using Cytoscape software [29]. Statistical comparison between tumor groups were done in GraphPad Prism v6 using a non-parametric Mann-Whitney test. Prognostic signatures were developed using R v3.2.4 and BRB Array Tools, developed by Dr. Richard Simon and BRB Array Tools Development Team [30]. Briefly, functional node activities were ranked according their p-values in a Kaplan-Meier analysis. Then, a Cox regression including a leave-one-out validation using 1,000 random permutations was used to validate the prognostic capability. P-values under 0.05 were considered statistically significant.

## Results

### Patient characteristics

Clinical characteristics of this patient cohort have been previously described [2, 3, 31]. Briefly, one hundred and six patients were enrolled into the study. They all had node positive disease, Her2 negative and all had received adjuvant chemotherapy and hormonal therapy in the case of ER+ tumors. Among ER+ tumors, 50 patients were characterized as ER-true and 21 were defined as TN-like (S1 Table) [2].

### Mass spectrometry analysis

Proteomics analyses from these samples have been previously described [2]. In summary, one hundred and two FFPE samples had enough protein to perform the MS analyses. After MS workflow, 96 samples provided useful protein expression data. After quality criteria, 1,095 proteins presented at least two unique peptides and detectable expression in at least 75% of the samples in at least one type of sample (either ER+ or TNBC).

### Directed networks

Using proteomics data, directed acyclic graphs (DAG) were performed. Altogether, it was possible to establish 789 edges of which 662 were guided and 127 are undetermined. These edges formed 303 components formed by different number of nodes or proteins. An overview of the number of nodes (proteins) included in each component is provided in Table 1.

**Table 1:** Characteristics of the components obtained from DAG.

**Number of nodes**	1	2	3	4	5	6	8	9	10	11	12	13	15	17	18	23	464
**Number of components**	188	62	13	18	6	4	2	1	1	1	1	1	1	1	1	1	1

Number of nodes = number of proteins contained in each component, Number of components = directed components obtained.

We characterized components from DAG analysis. Components including less than 9 nodes were dismissed because they were little informative. All components were named with the number of nodes included by the DAG analysis.

Afterwards, components were interrogated for biological function. Characteristics about all components are supplied in Table 2 and S1 File.

**Table 2:** Features of components obtained by DAG analysis.

Component	Number of nodes	Main function
**Component 23**	23	Membrane and mitochondria
**Component 18**	18	Cytoskeleton
**Component 17**	17	RNA binding
**Component 15**	15	Extracellular exosome
**Component 13**	13	Extracellular matrix
**Component 12**	12	RNA binding and translation
**Component 11**	11	Proliferation and oxphos
**Component 10**	10	Extracellular exosome
**Component 9**	9	RNA binding and splicing

### Component activity measurements

**Fig 1:**
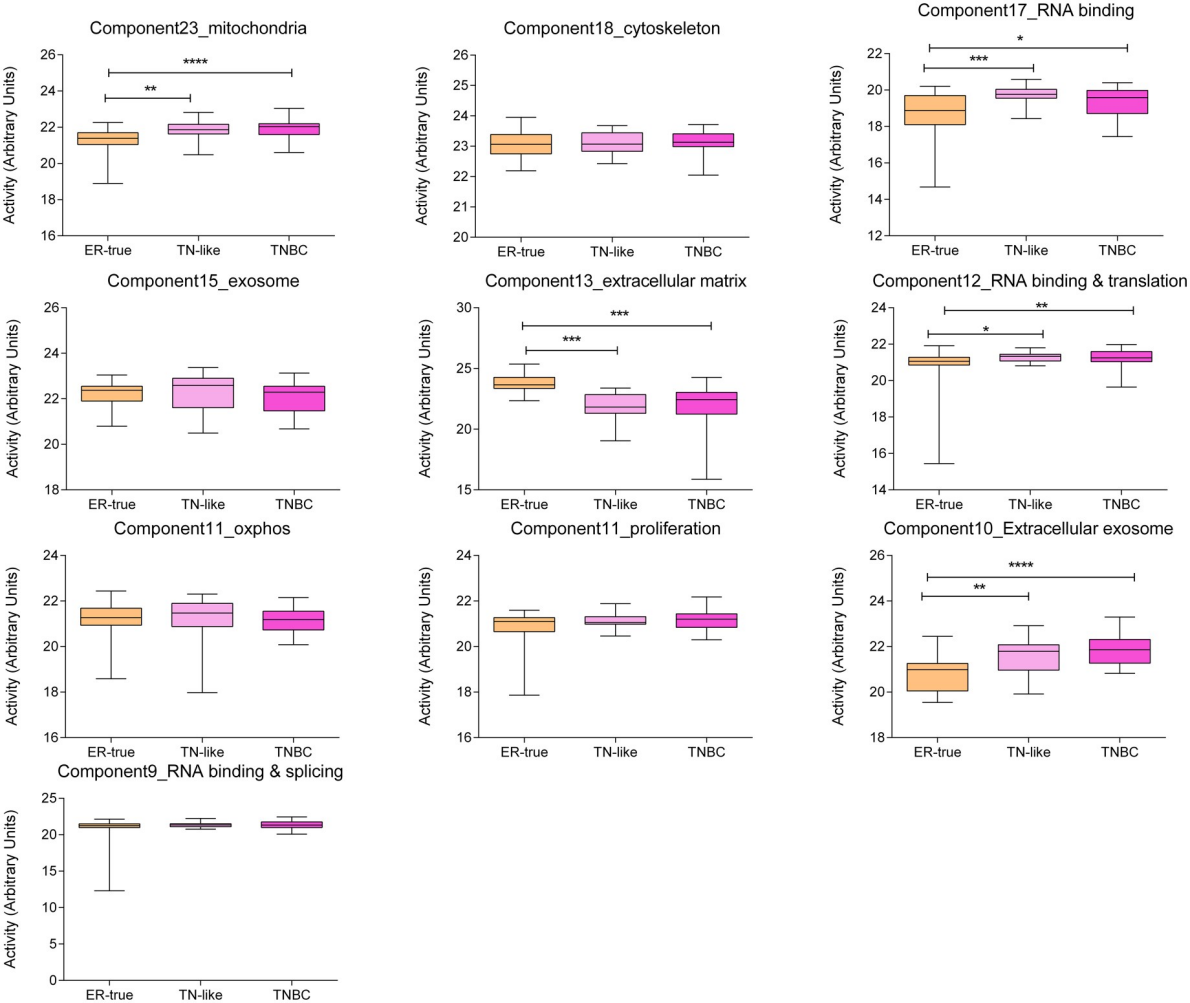
Component activity measurements for ER-true, TN-like and TNBC respectively.

Component activities were calculated for each node. There were significant differences between ER-true, TN-like and TNBC tumors in the component activity for component 23: mitochondria, component 17: RNA binding, component 13: extracellular matrix, and component 10: extracellular exosome (Fig 1).

### Component 13: Extracellular matrix

**Fig 2:**
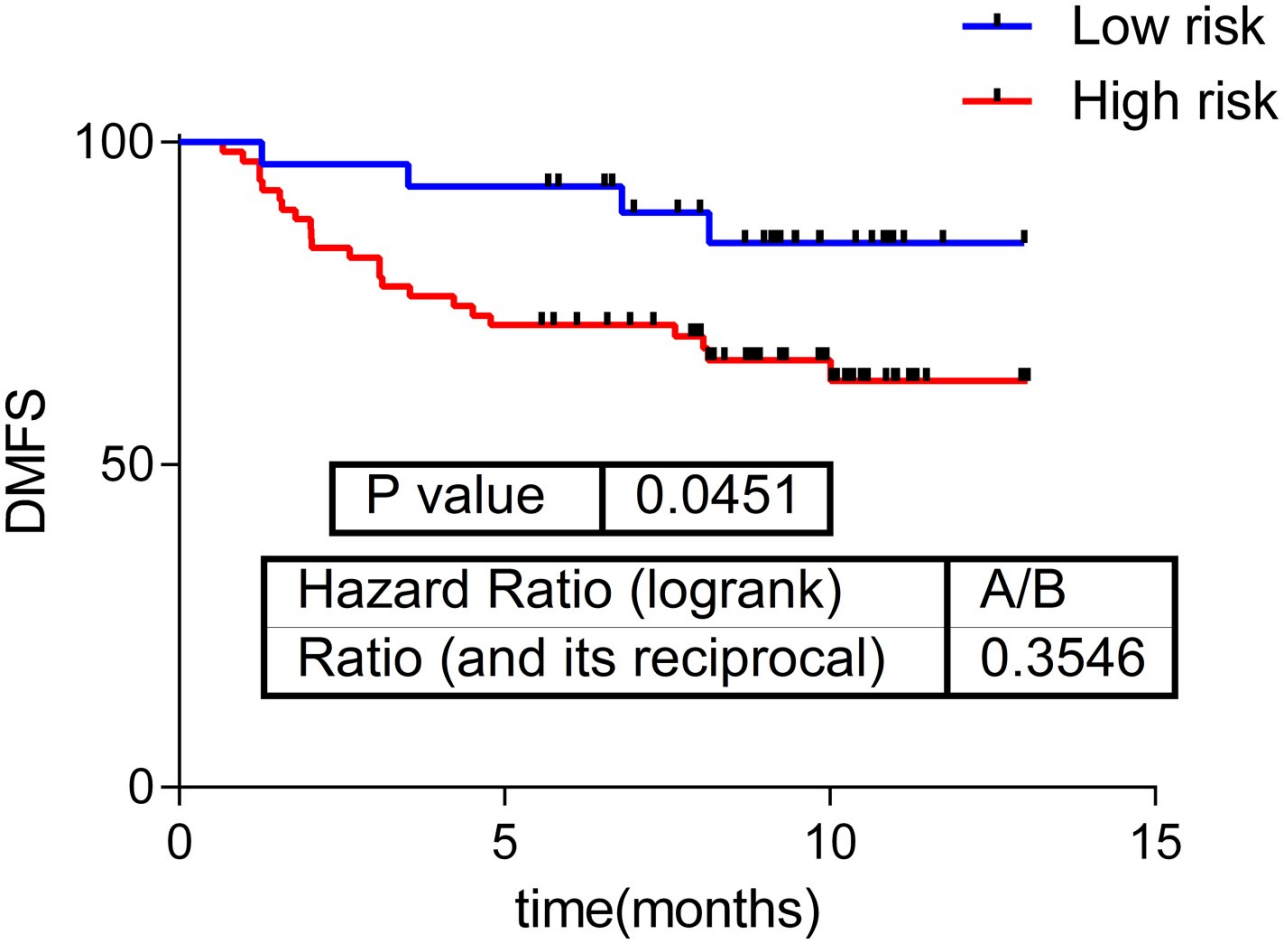
Component 13 activity prognostic value in the whole cohort.

Component 13 activity showed prognosis value in our series, splitting our population into a high and a low risk group and it can be used as a distant-metastasis free survival (DMFS) predictor (p = 0.045, HR = 0.35, 30–70%) (Fig 2). Interestingly, the predictor classified all the TN-like tumors and most of TNBC tumors into the high-risk group (Table 3).

**Table 3:** Number of patients classified by the DMFS predictor into a low or a high-risk group.

Subtype	Number in low-risk group	Number in high-risk group
**ER-true**	26	24
**TN-like**	0	21
**TNBC**	3	22

**Fig 3:**
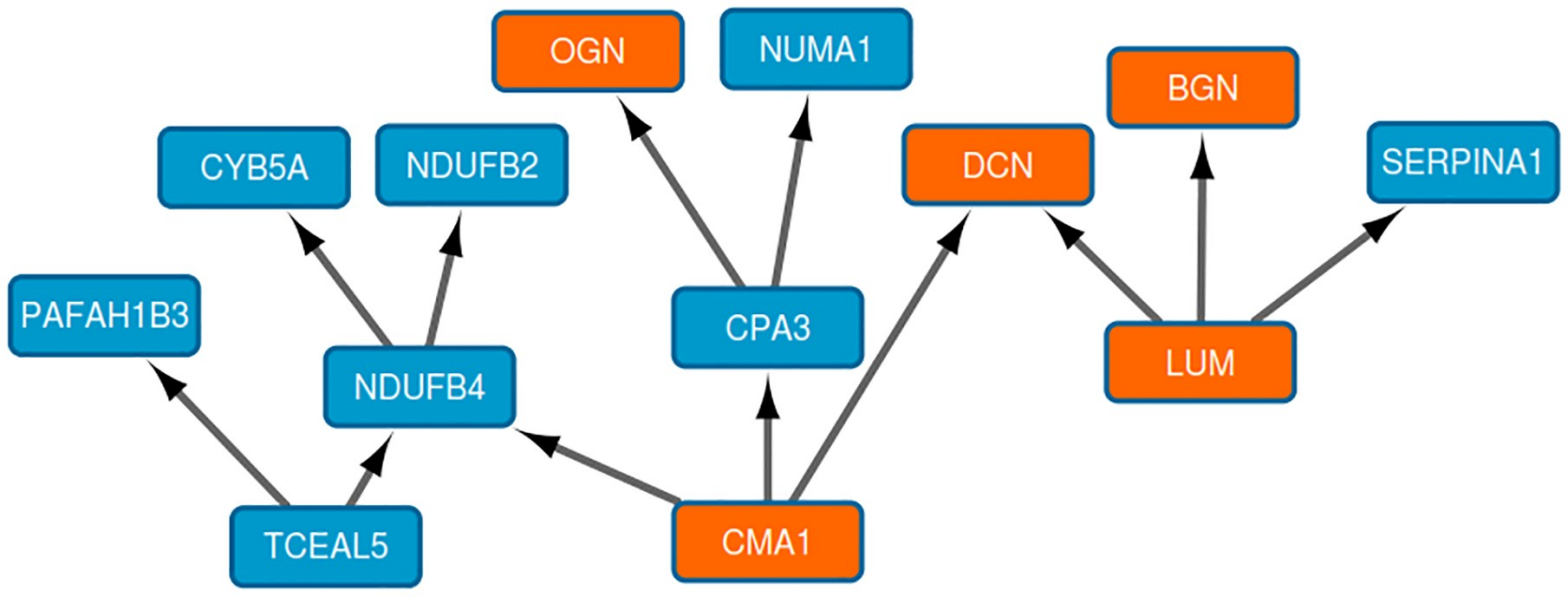
Component 13. Orange nodes: Proteins related to extracellular matrix ontology.

Component 13 contains thirteen proteins mainly related to extracellular matrix (Fig 3). The five proteins related by gene ontology analysis to extracellular matrix were OGN, BGN, LUM, CMA1, and DCN, all of them, with the exception of CMA1, belonged to small leucine-rich proteoglycan family of proteins.

**Fig 4:**
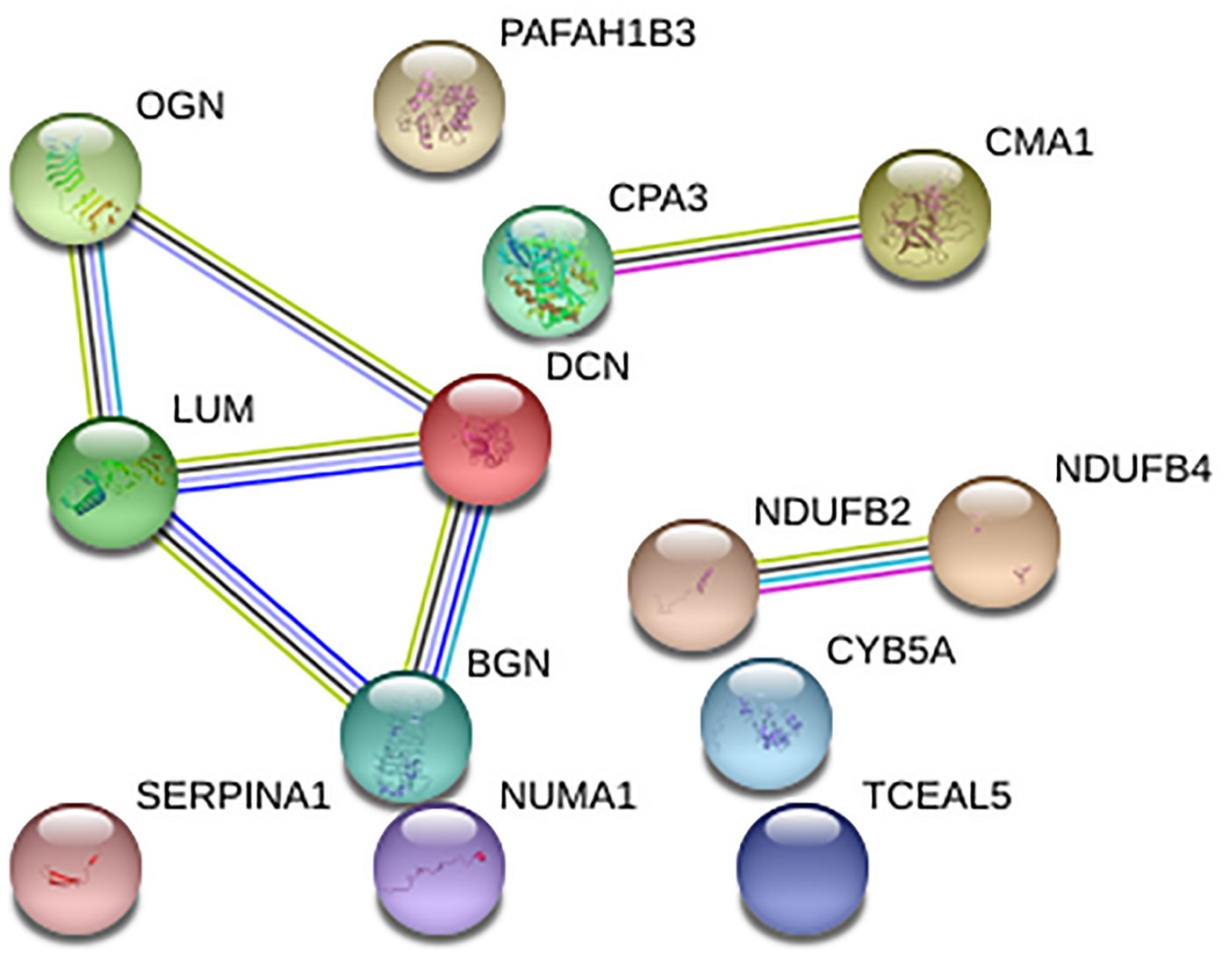
Network built with the proteins from Component13 using STRING.

Additionally, the existence of described connections was checked using STRING v11 (Fig 4). Interestingly, some of the connections suggested by BN analysis in Component 13, (NDUFB2 and NDUFB4; CPA3 and CMA1; and BGN, LUM, and DCN) were connected both in the DAG graph and in the STRING network, meaning that these interactions have been previously described.

## Discussion

In this study, we used proteomics and DAG to characterize relationships between proteins in breast cancer tumor samples. Unlike other approaches, such as Genes2FANS [32], our DAG method supplies directed relationships between proteins and a hierarchical structure. Traditionally, protein-protein interaction (PPI) networks, such as STRING, are based in relationships described in the literature. However, we built a directed network, i.e. a graph formed by edges with a direction, using protein expression data without other a priori information, so it was possible to propose new hypotheses about protein interactions. We used probabilistic graphical models (PGM) because they offer a way to relate many random variables with a complex dependency structure.

As it has been previously mentioned, arrows in directed networks indicate causality between two proteins. This approach allows making hypotheses about causal relationships between proteins and proposes a hierarchical structure. In some cases, an experimental relationship between two proteins connected in the directed network had been previously described. For instance, in component 18, it has been widely described that PIP binds AZGP1 in breast cancer [33]. Another example is component 11 which related COX5A, MT-CO2 and COX6C, all proteins of mitochondrial complex IV [34].

We demonstrated in previous works that non-directed graphs provided functional information [2, 3]. Interestingly, a functional structure also appeared in this type of networks. Component activities suggested differences in functions such as extracellular matrix and mitochondria. Differences in mitochondria between these subtypes have been previously described using non-directed PGMs [2, 3].

On the other hand, component 13, composed by thirteen proteins, five of them related to extracellular matrix, had prognostic value in our series. Of these five proteins, four of them belonged to the small leucine-rich proteoglycan (SLRP) family (lumican, biglycan, osteoglycin, and decorin). Biglycan (BGN) could promote migration in breast cancer [35]. It has been widely described the anti-metastatic role of decorin (DCN1) in breast cancer [36–38]. Lumican (LUM) significantly attenuated cell functional processes, including proliferation, migration and invasion [39]. In the same study, it was described that lumican modulates matrix effectors in MCF7 and MDAMB231 cells. Finally, osteoglycin (OGN) has been suggested as a biomarker of ECM in TNBC [40]. On the other hand, chymase 1 (CMA1) is secreted by mast cells and may play a role in angiogenesis [41]. It is also involved in extracellular matrix degradation. Higher levels of this protein were observed in Luminal subtype [42]. DCN, OGN, BGN, and LUM appeared also interconnected in the STRING network, so the DAG graph reflected protein interactions previously described. NDUFB2 and NDUFB4 were also connected in both networks.

Other proteins included in this component related to cancer are NUMA1, SERPINA1, and PAFAH1B3. Nuclear mitotic apparatus (NUMA1) is a structural component of the nuclear matrix. The encoded protein interacts with microtubules and plays a role in formation and organization of the mitotic spindle during cell division. It also modulates p-53 mediated transcription in cancer cells [43]. Serpin family A member 1 (SERPINA1) is a direct estrogen receptor target and a predictor of survival in breast cancer patients [44]. Platelet activating factor acetylhydrolase 1b catalytic subunit 3 (PAFAH1B3) encodes an acetylhydrolase that catalyzes the removal of an acetyl group from the glycerol backbone of platelet-activating factor. A study identified PAFAH1B3 as a key metabolic driver of breast cancer pathogenicity that is upregulated in primary human breast tumors and correlated with poor prognosis [45]. This enzyme may be dysregulated across many cancer types [46].

In previous studies we have used functional node activities from non-directed network to develop prognostic predictors [7]. Now, this approach is also validated in directed networks.

We used a mathematical method as DAG analysis and applied them to proteomics data of breast cancer tumors in order to infer causal relationships between these proteins. This method supplied some known relationships but also proposed new ones. Additionally, it associated proteins with a similar function. Therefore, it seems that it is a good approach to propose new hypotheses about mechanisms of action. Moreover, it was possible to associate the results obtained by DAG analysis with prognosis and built a prognostic signature. As far we know, this is the first time that this type of analysis is applied to clinical data and is associated with clinical outcome.

Our study has some limitations. Proteomics provides complementary information to other techniques such as genomics. However, an improvement in the number of detected proteins is still necessary. On the other hand, breast cancer clinical scenario is far more complex, and stratified analyses (by molecular or clinical subtypes, for example) could provide more complex and insightful information. Finally, all these mathematical approaches (and others), despite being useful by themselves, should be combined to obtain more information about the clinical scenario analyzed, as long as it seems that different analyses provide different and complementary information from the same data.

To sum up, in this study, we used proteomics and directed networks to characterize relationships between proteins in breast cancer tumors. This approach reflected some previously described interactions and it could be used to propose new hypotheses and mechanisms of action.

## Supporting information

S1 File Components obtained by DAG analysis. (DOCX)

S1 Table Patient characteristics. (DOCX)
