# Supplementary figures and images for: Bayesian networks established functional differences between breast cancer subtypes
Source: PLoS One. 2020 Jun 11;15(6):e0234752. doi: 10.1371/journal.pone.0234752 (PMC7289386; doi:10.1371/journal.pone.0234752)

Component 23


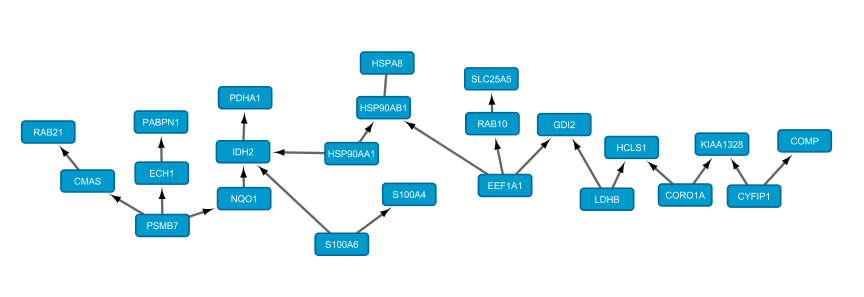


Component 18


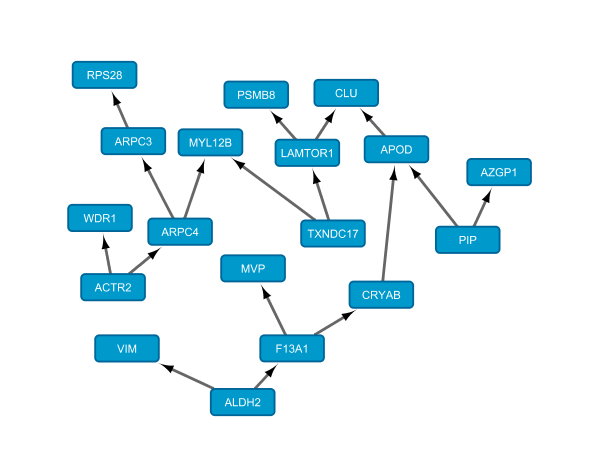


Component 17


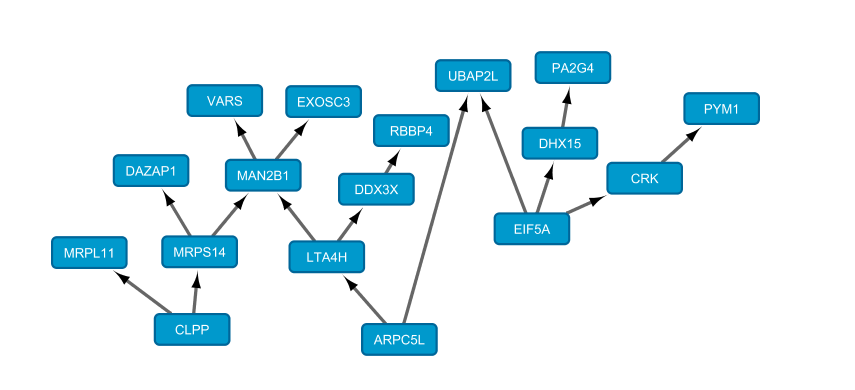


Component 15


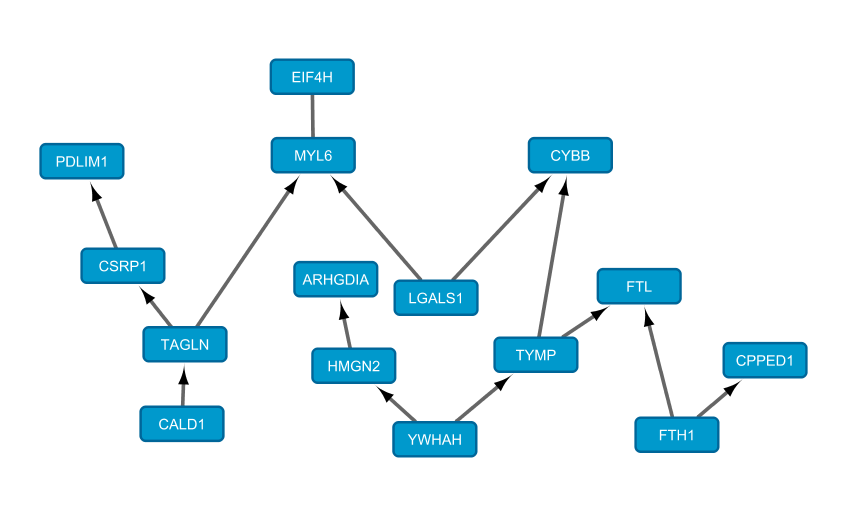


Component 12


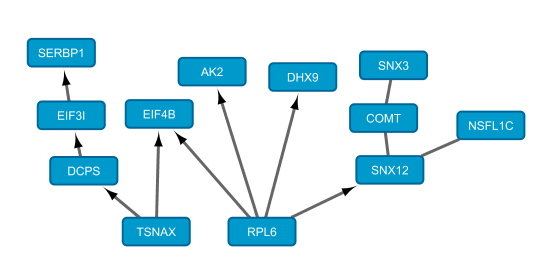


Component 11


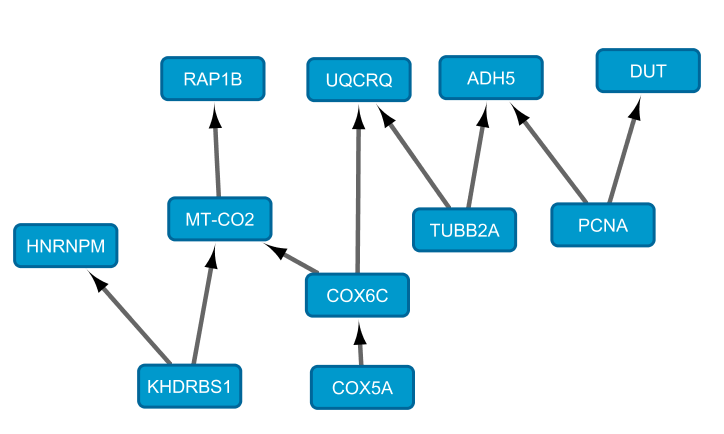


Component 10


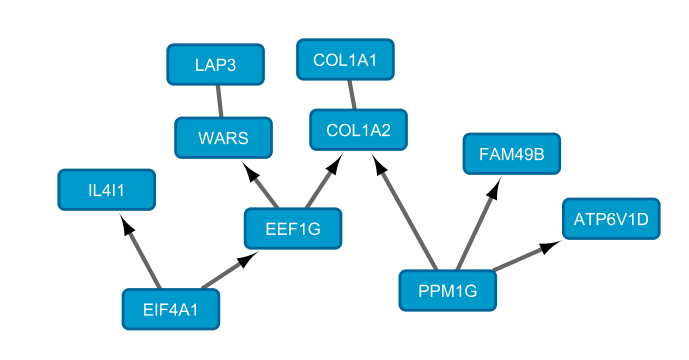


Component 9


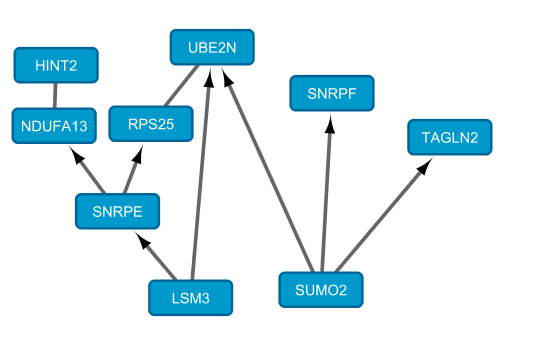

Supplement: S1 File — (DOCX) [file pone.0234752.s001.docx]
