# Supplementary material for: Bayesian networks established functional differences between breast cancer subtypes
Source: PLoS One. 2020 Jun 11;15(6):e0234752. doi: 10.1371/journal.pone.0234752 (PMC7289386; doi:10.1371/journal.pone.0234752)
Supplement: S1 Table — (DOCX) [file pone.0234752.s002.docx]

|  | All | TNBC | ER+ | TN-like | ER-true |
| --- | --- | --- | --- | --- | --- |
| Number of patients | 106 | 26 | 80 | 21 | 50 |
| Age at diagnosis (median and range) | 54.6 (32-83) | 61.2 (37-78) | 54.2 (32-83) | 54.04 (35-71) | 53.9 (32-83) |
| Age at diagnosis (mean) | 55.2 | 58.5 | 54.1 | 53.83 | 53.5 |
| Tumor Size |  |  |  |  |  |
| T1 | 33 (31%) | 5 (19%) | 28 (35%) | 7 (33%) | 17 (34%) |
| T2 | 61 (58%) | 19 (73%) | 42 (53%) | 13 (62%) | 25 (50%) |
| T3 | 10 (9%) | 2 (8%) | 8 (10%) | 1 (5%) | 6 (12%) |
| T4 | 1 (1%) | 0 (0%) | 1 (1%) | 0 (0%) | 1 (2%) |
| Multifocal | 1 (1%) | 0 (0%) | 1 (1%) | 0 (0%) | 1 (2%) |
| Tumor Grade |  |  |  |  |  |
| G1 | 12 (11%) | 0 (0%) | 12 (15%) | 3 (14%) | 7 (14%) |
| G2 | 33 (31%) | 4 (15%) | 29 (36%) | 5 (24%) | 20 (40%) |
| G3 | 41 (39%) | 20 (77%) | 21 (26%) | 10 (48%) | 10 (20%) |
| Unknown | 20 (19%) | 2 (8%) | 18 (23%) | 3 (14%) | 13 (26%) |
| Lymph node status |  |  |  |  |  |
| N0 | 0 (0%) | 0 (0%) | 0 (0%) | 0 (0%) | 0 (0%) |
| N1 | 71 (67%) | 17 (65%) | 54 (68%) | 13 (62%) | 34 (68%) |
| N2 | 35 (33%) | 9 (35%) | 26 (32%) | 8 (38%) | 16 (32%) |
